# Off-target effects and clinical outcome in metastatic colorectal cancer patients receiving regorafenib: The TRIBUTE analysis

**DOI:** 10.1038/srep45703

**Published:** 2017-04-05

**Authors:** Riccardo Giampieri, Michela del Prete, Tiziana Prochilo, Marco Puzzoni, Valeria Pusceddu, Fabiana Pani, Elena Maccaroni, Roberta Mascia, Maria Giuditta Baleani, Tania Meletani, Rossana Berardi, Anna Maria Lanzillo, Stefano Mariotti, Alberto Zaniboni, Stefano Cascinu, Mario Scartozzi

**Affiliations:** 1 Medical Oncology, University Hospital and Polytechnic University of the Marche, Ancona Italy; 2 Fondazione Poliambulanza Hospital, Brescia, Italy; 3 Medical Oncology, University Hospital and University of Cagliari, Cagliari, Italy; 4 Endocrinology Unit, University Hospital and University of Cagliari, Cagliari, Italy; 5 Medical Oncology, Azienda Ospedaliera “Brotzu” Cagliari, Italy; 6 Medical Oncology, University Hospital and University of Modena and Reggio Emilia, Modena, Italy

## Abstract

Regorafenib is an orally administered multikinase inhibitor indicated for the treatment of heavily pretreated metastatic colorectal cancer patients with good performance status, albeit less than 50% treated patients achieve disease stabilisation or better at the first radiological evaluation. In addition to that a particularly broad spectrum of toxicities (experienced as G3 or more NCI CTCAE graded by 50% of patients treated) have led to reconsider its widespread use in the majority of patients. We retrospectively collected data about the magnitude of off-target effects experienced during the first 8-weeks of regorafenib monotherapy and analysed their correlation with overall survival, progression free survival and disease control rate. Our findings suggest that skin rash (Exp (B): 0.52, p = 0.0133) or hypothyroidism (Exp (B): 0.11, p = 0.0349) were significantly correlated with improved overall survival at multivariate regression analysis. It was also demonstrated a statistically significant role of diarrhea as predictor of improved survival but its independent prognostic role was lost at multivariate analysis (Exp (B): 0.63, p = 0.162). This is the first analysis showing a potential correlation between the onset of these forms of side effects and regorafenib efficacy, however sample size limitations and the retrospective nature of our analysis prevent us from drawing definitive conclusions.

Regorafenib represents a relevant treatment opportunity for patients with metastatic colorectal cancer (mCRC) failing previous therapy with fluoropyrimidine, oxaliplatin, irinotecan-based chemotherapy, anti-VEGF therapy and, if RAS wild type, anti-EGFR therapy^1^,^2^,^3^,^4^,^5^. The most common adverse effects observed with the use of regorafenib include hand and foot skin reaction (HFSR) (17%), skin rash (6%), fatigue (10%), diarrhoea (7%) and hypertension (7%)^2^.

Some of these adverse reactions, although not fatal, can be debilitating causing both physical and emotional discomfort^6^. In addition, not all subgroups of patients seem to benefit from treatment with regorafenib and therefore a non-negligible proportion of patients are exposed to unnecessary toxicity without deriving any clinical advantage. Many clinical and biological variables have been analysed in order to better identify patients more likely to achieve an improved clinical outcome from this treatment. Unfortunately, to date, no predictive marker has been validated for the clinical practice^7^,^8^,^9^,^10^. An interesting area of research in this setting is also represented by the potential role of off-target effects of different targeted agents to affect clinical outcome. In patients diagnosed with different tumour types receiving oral multikinase inhibitors with a pharmacological profile partially overlapping regorafenib (e.g. sunitinib, sorafenib, pazopanib) off-target adverse effects have been analysed as possible early markers of response and clinical benefit. In advanced hepatocellular carcinoma patients receiving sorafenib the occurrence of early HFSR correlated in fact with a prolonged time to progression (TTP) and an improved overall survival (OS)^11^,^12^,^13^. Similarly, arterial hypertension has been correlated with improved OS during treatment with sorafenib, sunitinib and pazopanib in different malignancies^14^. Sorafenib-induced diarrhoea has been associated with improved OS^15^. In this view also thyroid dysfunction has been evaluated in patients treated with sunitinib. In renal cell carcinoma patients receiving sunitinib the development of antithyroid peroxidase (TPOAb) autoantibodies along with severe hypothyroidism was associated with a longer progression free survival (PFS)^16^. In the TRIBUTE (Toxicity during Regorafenib Induction and Benefit Under Treatment Extension) analysis we evaluated the potential role of off target effects as early surrogate markers of clinical outcome in patients with colorectal cancer treated with regorafenib with the final aim to improve the early identification of patients more likely to benefit from such a treatment approach and optimise adverse events management.

## Results

A total number of 144 patients were included in our analysis. In the global population 9 (6%) patients achieved partial response, 37 (26%) patients had stable disease at their first radiological evaluation. Eighty-six (60%) patients progressed under treatment. In 12 (8%) patients radiological evaluation of response was not performed (Table 1). Median progression free survival was 2.8 months (95%CI: 2.557–3.148) and median overall survival was 6 months (95%CI: 4.0–9.213). One hundred and twenty-four patients (86%) had already progressed and 89 patients (62%) had already died at the time of analysis. Forty-one patients (28%) showed HFSR, 21 patients (15%) had diarrhea, 29 patients (20%) had hypertension, 38 patients (26%) had skin rash, 65 patients (45%) had fatigue, 18 patients (12%) had increased AST/ALT, 15 patients (10%) had increased bilirubin, 13 patients (9%) showed hypothyroidism.

### Off-target effects and Clinical Outcome

#### HFSR

**Figure 1:**
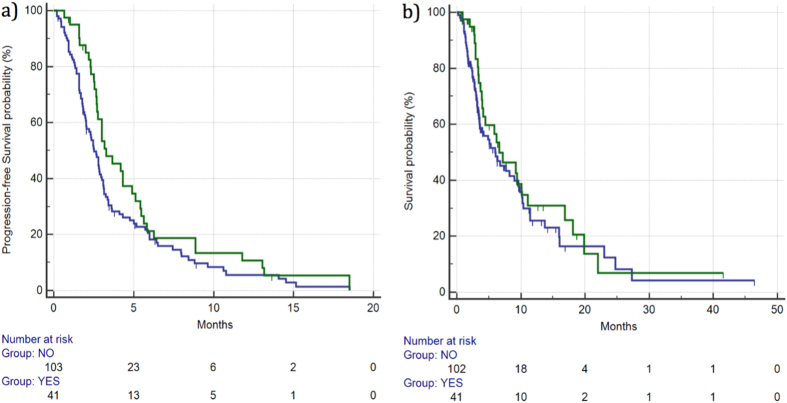
Median PFS (**a**) and OS (**b**) for patients who developed ≥ grade 2 HFSR (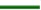) *vs* patients with grade 0–1 HFSR (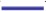). Median PFS was 3.27 *vs* 2.52 months (HR: 0.73, 95%CI: 0.51–1.04, p = 0.10), respectively, whereas median OS was 6.59 *vs* 5.96 months (HR: 0.80, 95%CI: 0.52–1.25, p = 0.35), respectively.

Disease control rate (DCR) for patients with grade ≥2 HFSR was 47% *vs* 25% for the remaining patients. This difference was statistically significant (p = 0.0269). Median PFS for patients with grade ≥2 HFSR *vs* the remaining patients was 3.2 *vs* 2.5 months (HR: 0.73, 95%CI: 0.51–1.04, p = 0.10), respectively. (Figure 1). Median OS for patients with grade ≥2 HFSR *vs* the remaining patients was 6.59 *vs* 5.96 months (HR: 0.80, 95%CI: 0.52–1.25, p = 0.35), respectively. (Figure 1) (Table 2).

#### Skin Rash

**Figure 2:**
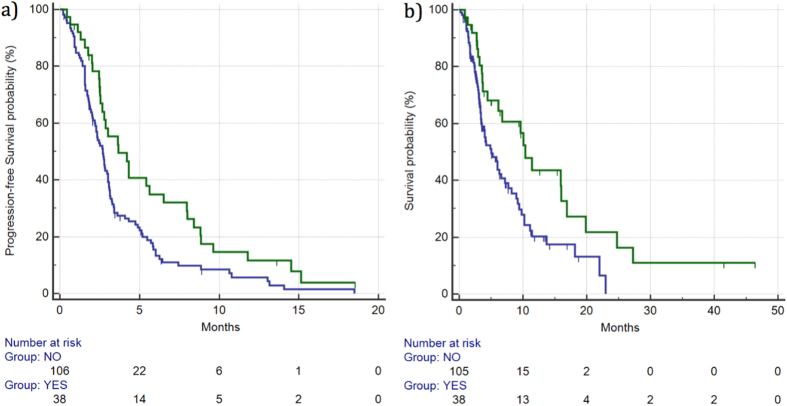
Median PFS (**a**) and OS (**b**) for patients who developed ≥grade 2 skin rash (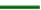) *vs* patients with grade 0–1 skin rash (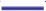). Median PFS was 3.67 *vs* 2.68 months (HR: 0.60, 95%CI: 0.42–0.86, p = 0.0096), respectively, whereas median OS was 10.36 vs 5.04 months (HR: 0.54, 95%CI: 0.36–0.84, p = 0.0085), respectively.

DCR for patients showing skin rash grade ≥2 was 55% *vs* 21% for the remaining patients. This difference was statistically significant (p = 0.000387). Median PFS for patients with grade ≥2 skin rash *vs* the remaining patients was 3.67 *vs* 2.68 months (HR: 0.60, 95%CI: 0.42–0.86, p = 0.0096), respectively. (Figure 2). Median OS for patients with grade ≥2 skin rash *vs* the remaining patients was 10.36 *vs* 5.04 months (HR: 0.54, 95%CI: 0.36–0.84, p = 0.0085), respectively. (Figure 2) (Table 2).

#### Hypertension

**Figure 3:**
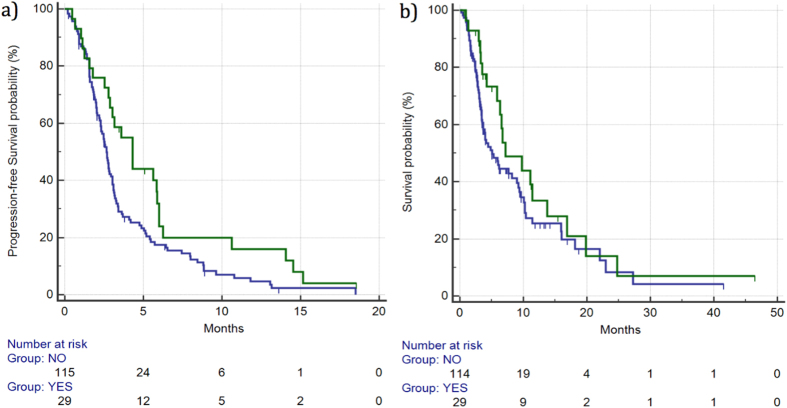
Median PFS (**a**) and OS (**b**) for patients who developed ≥grade 2 hypertension (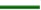) vs patients with grade 0–1 hypertension (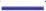). Median PFS was 4.32 *vs* 2.65 months (HR: 0.61, 95%CI: 0.41–0.89, p = 0.0197), respectively, whereas median OS was 7.18 *vs* 5.04 months (HR: 0.71, 95%CI: 0.45–1.13, p = 0.188), respectively.

DCR was 48% *vs* 27% (p = 0.0526), respectively, for patients showing grade ≥2 hypertension *vs* the remaining patients. Median PFS for patients who developed grade ≥2 hypertension was 4.32 months *vs* 2.65 months for the remaining patients (HR: 0.61, 95%CI: 0.41–0.89, p = 0.0197) (Fig. 3).

Median OS for patients who developed grade ≥2 hypertension *vs* those who did not was 7.18 *vs* 5.04 months (HR: 0.71, 95% CI: 0.45–1.13, p = 0.188), respectively. (Figure 3) (Table 2).

#### Diarrhea

**Figure 4:**
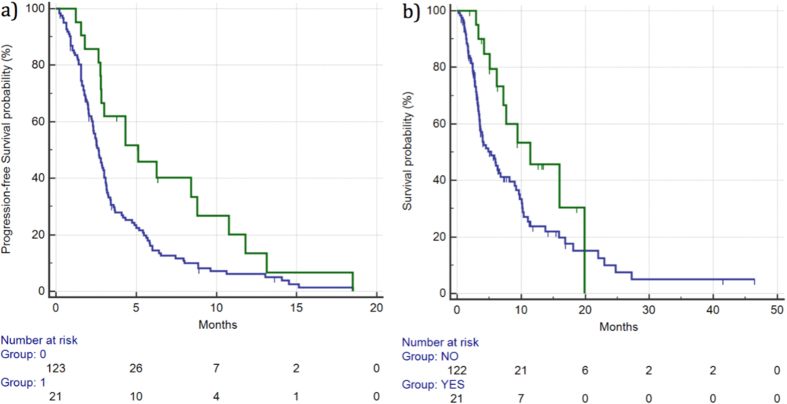
Median PFS (**a**) and OS (**b**) for patients who developed ≥grade 2 diarrhea (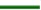) *vs* patients with grade 0–1 diarrhea (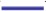). Median PFS was 5.11 *vs* 2.65 months (HR: 0.54, 95%CI: 0.36–0.81, p = 0.0130), respectively, whereas median OS was 11.41 *vs* 5.27 months (HR: 0.53, 95%CI: 0.32–0.88, p = 0.0440), respectively.

DCR was 53% vs 27% (p = 0.0489), respectively, for patients experiencing grade ≥2 diarrhea *vs* the remaining patients. Median PFS for patients who experienced grade ≥2 diarrhea was 5.11 *vs* 2.65 months for the remaining patients (HR: 0.54, 95%CI: 0.36–0.81, p = 0.0130). (Figure 4). Median OS for patients who experienced grade ≥2 diarrhea was 11.41 *vs* 5.27 months for the remaining patients (HR: 0.53, 95%CI: 0.32–0.88, p = 0.0440). (Figure 4) (Table 2).

#### Hypothyroidism

**Figure 5:**
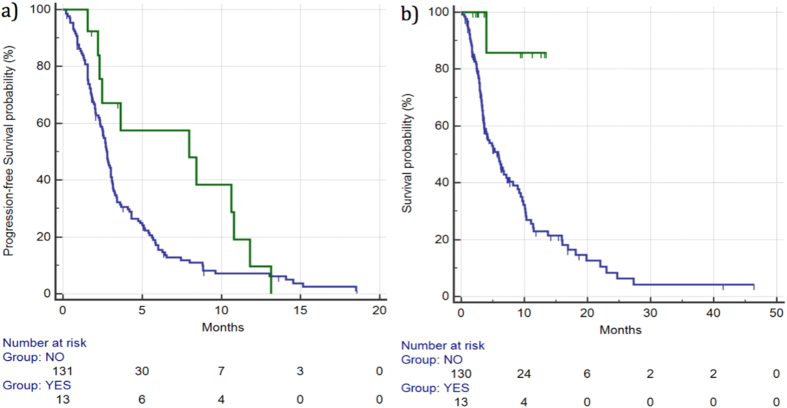
Median PFS (**a**) and OS (**b**) for patients who developed ≥grade 2 hypothyroidism (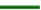) *vs* patients with grade 0–1 hypothyroidism (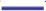). Median PFS was 7.96 *vs* 2.75 months (HR: 0.56, 95%CI: 0.34–0.93, p = 0.0635), respectively, whereas median OS was 7.96 *vs* 2.75 months (HR: 0.56, 95%CI: 0.34–0.93, p = 0.0635), respectively.

DCR for patients showing grade ≥2 hypothyroidism *vs* the remaining patients was 71% vs 28% (p = 0.03), respectively. Median PFS for patients who experienced grade ≥2 hypothyroidism was 7.96 *vs* 2.75 months for the remaining patients (HR: 0.56, 95%CI: 0.34–0.93, p = 0.0635) (Fig. 5).

Median OS for patients showing grade ≥2 hypothyroidism was not reached *vs* 5.83 months for the remaining patients (HR: 0.10, 95%CI: 0.05–0.19, p = 0.0042) (Fig. 5) (Table 2).

#### Fatigue

DCR was 32% *vs* 30% (p = 1.0), respectively, for patients experiencing grade ≥2 fatigue *vs* the remaining patients. Median PFS for patients who developed grade ≥2 fatigue *vs* the remaining patients was 2.85 *vs* 2.68 months (HR: 0.93, 95%CI: 0.66–1.32, p = 0.716), respectively. Median OS was also not significantly different between the two groups of patients, with median OS 7.18 *vs* 6.03 months, respectively, for patients who developed grade ≥2 fatigue *vs* the remaining patients (HR: 0.76, 95%CI: 0.50–1.14, p = 0.195) (Table 2).

#### AST/ALT increase

DCR was 43% *vs* 29% (p = 0.22), respectively, for patients showing grade ≥2 AST/ALT increase *vs* the remaining patients. Median PFS for patients showing grade ≥2 AST/ALT increase *vs* the remaining patients was 3.01 *vs* 2.78 months (HR: 1.11, 95%CI: 0.65–1.89, p = 0.68), respectively.

Median OS for patients who experienced grade ≥2 AST/ALT increase *vs* the remaining patients was 4.13 *vs* 6.32 months (HR: 1.60, 95%CI: 0.83–3.10, p = 0.08), respectively (Table 2).

#### Bilirubin increase

DCR was 23% *vs* 32% (p = 0.75), respectively, for patients showing grade ≥2 bilirubin increase *vs* the remaining patients. Median PFS for patients showing grade ≥2 bilirubin increase *vs* the remaining patients was 2.82 *vs* 2.78 months (HR: 1.42, 95%CI: 0.75–2.69, p = 0.20), respectively. Median OS was not significantly worse in patients showing grade ≥2 bilirubin increase *vs* the remaining patients (8.98 *vs* 6.03 months, HR: 1.21, 95%CI: 0.63–2.33, p = 0.52, respectively) (Table 2).

### Other tested variables

#### ECOG PS

Forty-one patients (28%) showed an ECOG ≥1 whereas the remaining 103 patients (72%) were ECOG 0. DCR in ECOG 0 *vs* ECOG ≥1 was 29% *vs* 17% (p = 0.10), respectively. No statistically significant differences were observed for PFS and OS. In particular, median PFS for ECOG 0 *vs* ECOG ≥1 patients was 3.01 *vs* 2.69 months (HR: 0.88, 95%CI: 0.60–1.30, p = 0.52), respectively, whereas median OS for ECOG 0 *vs* ECOG ≥1 was 6.75 *vs* 6.16 months (HR: 0.70, 95%CI: 0.44–1.10, p = 0.10), respectively.

#### Gender

Eighty-two (57%) patients were male whereas the remaining 62 (43%) were female. DCR was 30% *vs* 9% for male *vs* female patients (p = 0.17), respectively. Median PFS for male *vs* female patients was 3.01 *vs* 2.65 months, HR: 0.77, 95%CI: 0.54–1.10, p = 0.13), respectively. Median OS for male *vs* female patients was 7.67 *vs* 5.27 months (HR: 0.81, 95%CI: 0.53–1.23, p = 0.31), respectively.

#### Age

Patients’ median age was 62 (range 32–80). Age (<70 *vs* ≥70 years) did not have a statistically significant impact on patients’ clinical outcome. Twenty-one patients (14%) were ≥70 years old whereas the remaining 123 (86%) were <70 years old. DCR in the <70 *vs* ≥70 years old groups was 28 and 14% (p = 0.28), respectively. Median PFS was 2.88 *vs* 2.29 months (HR: 1.06, 95%CI: 0.55–2.03, p = 0.85), respectively, for <70 *vs* ≥70 years old patients. Median OS was 5.83 vs 4.45 months (HR: 0.81, 95%CI: 0.37–1.75, p = 0.62), respectively, for <70 vs ≥70 years old patients.

#### RAS mutational status

Eighty-six (60%) patients were RAS mutant whereas the remaining 58 (40%) were RAS WT. We did not observe any significant differences in terms of DCR, OS and PFS between RAS WT patients *vs* RAS mutant.DCR was 34% for RAS WT patients while DCR was 20% for RAS mutant patients (p = 0.053), respectively. Median PFS for RAS WT patients *vs* RAS mutant was 2.85 *vs* 2.78 months (HR: 0.86,95%CI: 0.54–1.35, p = 0.50), respectively. Median OS for RAS WT patients *vs* RAS mutant was 5.54 *vs* 6.45 months (HR: 1.06, 95%CI: 0.64–1.73, p = 0.81), respectively.

#### Regorafenib dose at the time of progression

At the time of disease progression 57 patients (40%) were on full dose regorafenib (160 mg/day), 67 patients (46%) were on 120 mg/day regorafenib and the remaining 18 patients (12%) were receiving regorafenib 80 mg/day. Median PFS for 160 mg/day, 120 mg/day and 80 mg/day cohorts was 2.85, 2.95 and 3.14 months (p = 0.39), respectively. Median OS for 160 mg/day, 120 mg/day and 80 mg/day cohorts was 5.13, 6.27 and 5.27 months (p = 0.62), respectively.

### Multivariate analysis results

Multivariate analysis for PFS was performed by including in the model only those variables who were correlated with a stratistically significant impact on PFS in univariate analysis, namely skin rash development, hypertension and diarrhea.

All these factors maintained their independent roles as predictors of different PFS, respectively skin rash (cox-regression exponential [Exp (B)]: 0.59, p = 0.0112), hypertension (Exp (B): 0.57, p = 0.0115) and diarrhea (Exp (B): 0.47, p = 0.0042).

Multivariate analysis for OS was performed by including only those variables that resulted to be correlated with a significantly different OS at univariate analysis, namely skin rash, diarrhea, hypothyroidism At multivariate analysis for OS only skin rash (Exp (B): 0.52, p = 0.0133) and hypothyroidism (Exp (B): 0.11, p = 0.0349) maintained their independent role whereas diarrhea (Exp (B): 0.63, p = 0.162) lost its role as independent predictor of different OS.

**Figure 6:**
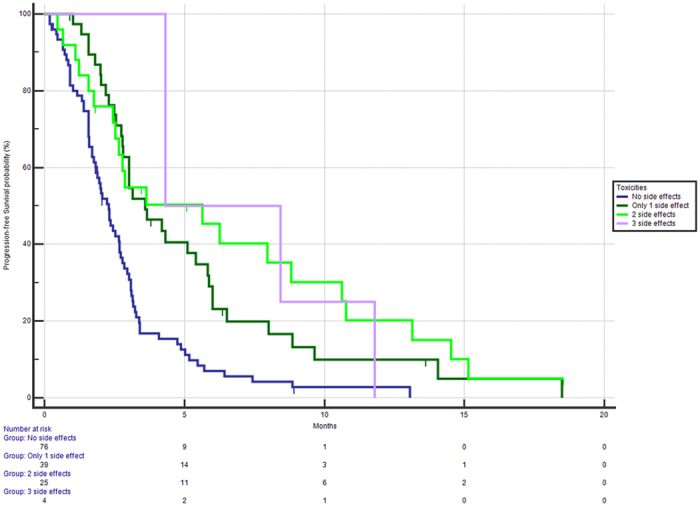
Median PFS stratified on the basis of having 0 (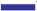), 1 (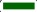), 2 (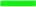) or 3 (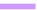) side-effects related to Regorafenib treatment. Median PFS were respectively 2.25 vs 3.60 vs 5.63 vs 4.32 months, p of the whole group <0.0001).

On the basis of the independent prognostic roles of each single toxicity identified as potential indicator of different PFS or OS, looking closely at patients who had more than one side-effects, a cumulative effect could be seen: in particular, when we looked at the PFS for patients who experienced all 3 side-effects (4/144, 3%) compared with those patients who just experienced 2 side-effects (25/144, 17%) or those with only 1 side-effect (39/144, 27%), a strikingly improved PFS was seen with each additional side-effect (HR for PFS respectively 0.36 for 3 side-effects vs none, 0.40 for 2 side-effects vs none, 0.48 for only 1 side-effects vs none, Fig. 6).

## Discussion

Since its introduction in 2013, fine-tuning of regorafenib-based therapy has always been a challenging issue. If on the one hand regorafenib is in fact able to improve the therapeutic possibilities for pre-treated metastatic colorectal cancer patients, on the other hand a non-negligible proportion of patients are exposed to toxicity without deriving any clinical benefit. Patients enrolled in the CORRECT and CONCUR^2^,^3^ trials have experienced as much as a 50% risk of severe (G3 NCI CTCAE or more) toxicity and an even more increased risk of less severe combined toxicities. Nonetheless, recent observational studies such as the CONSIGN and REBECCA trials^4^,^5^ have suggested that efficacy results are also reproducible in patients treated outside the setting of a randomised clinical trial. Unfortunately, along with the efficacy profile the toxicity profile was also unchanged and difficult to manage. The proper management of adverse events then becomes a relevant issue, particularly if we consider that as many as 50% of patients are not expected to benefit from this drug and that most patients are more likely to experience disease stabilisation, rather than a typical objective response. Several Authors have suggested a correlation between toxicity (off-target effects) and clinical outcome in different tumours types treated with different anti-neoplastic agents. We previously demonstrated a possible link between hypertension and clinical outcome for patients treated with first-line FOLFIRI and the anti-angiogenic monoclonal antibody bevacizumab^17^. Subsequently other Authors^18^,^19^ confirmed the correlation between increased arterial blood pressure during bevacizumab therapy and clinical outcome for patients with metastatic colorectal cancer. This correlation was also evident in other cancer types, such as metastatic breast cancer^20^. Similarly, in the present analysis hypertension was able to influence progression free survival, however this did not translate into an independent role for overall survival thus suggesting that hypertension might not be as relevant as other factors when a multi tyrosine kinase inhibitor, such as regorafenib, is used. Due to the broad range of pharmacological activity, regorafenib may induce skin-based toxicities such as skin rash or the more common hand-foot skin reaction. These peculiar toxicities are also common with other oral tyrosine kinase inhibitors, such as sorafenib, and require a careful clinical management to both lessen patients’ discomfort and maintain compliance to treatment. As with other forms of skin toxicities, in the past few years several analyses have been published regarding the correlation between targeted agents inducing skin toxicity and outcome. In particular, both panitumumab and cetuximab have a characteristically favorable response profile in patients experiencing severe skin rash^21^,^22^,^23^. Recently, a study on the potential correlation between favorable outcome and the occurrence of HFSR in patients with HCC treated with sorafenib^13^ has been published. Similarly to our findings, patients experiencing grade ≥2 skin toxicity with sorafenib therapy had the greatest probability of achieving an improved disease control. Granito *et al*.^12^ also recently published an extensive review on the prognostic role of different toxicities on outcome for patients with HCC treated with sorafenib. Interestingly, the occurrence of HFSR with sorafenib was one of the factors more frequently correlated with improved survival. In our analysis, however, skin rash was significantly correlated with improved overall survival and progression free survival, whereas only a not statistically significant trend was observed for HFSR. This discrepancy might be at least in part related to the increased use of prophylactic measures to counteract this frequent and unpleasant side effect. In fact, most patients treated pre-emptively usually experience less severe grades of HFSR and usually not as early as untreated patients.

Our analysis suggested that other relevant off-target effects (such as diarrhea and hypothyroidism) might correlate to clinical activity in patients treated with regorafenib. Although to date this is the first analysis showing a potential correlation between the onset of these forms of side effects and regorafenib efficacy, a similar correlation can be observed once again in HCC patients receiving sorafenib. In particular, Di Costanzo *et al*.^24^ recently published results of a cohort analysis comparing the outcome of patients who experienced these forms of toxicities while on treatment and those who experienced the same “side-effects” prior to treatment. The Authors found that, only in the population of patients on treatment, the occurrence of diarrhea and hypertension were related to improved survival. On the other hand, hypothyroidism results were in line with previous observations of renal cell carcinoma patients receiving sunitinib.

Sample size limitations, the absence of a control group and the retrospective nature of our analysis prevent us from drawing definitive conclusions about the role of off-target effects correlated with clinical outcome during treatment with regorafenib, but at least our paper is able to confirm the fact that, if managed properly, toxicity from regorafenib-based therapy should not be viewed like a factor that should discourage medical oncologists in prescribing and continuing treatment. In our analysis only skin rash was independently correlated with both PFS and OS, whereas hypertension and diarrhea independently correlated with PFS and hypothyroidism correlated with OS. Strange as it may seem, these effects (particularly diarrhea, skin rash and hypothyroidism) seem to point out at some mechanism of action that, differently from what you would expect from an antiangiogenic drug, seems focused on the immune system.

Even though it should be tested in further analyses, that might be an intriguing field of further development: indeed, among its many targets, regorafenib also binds (and should block) both RET and KIT. These two targets are particularly well-known proto-oncogenes, with a correlation with different types of cancers such as the thyroid carcinomas for the former (and that might explain why hypothyroidism occurs) while the latter is involved in the pathogenesis of a series of different human cancers and in processes of hemopoiesis, T cell differentiation and lymphoid progenitor cell differentiation (and that might explain why the hypothesized auto-immune-like mechanism). It is then with some concern that suggestions^25^ regarding a potential usefulness in managing toxicities from regorafenib-based therapy with the use of corticosteroids, based on this hypothesis, should be advised, that is due the potential immunodepressant activity of corticosteroids.

This factor might explain why, also in regorafenib treated patients, lymphocyte count and neutrophile-to-lymphocyte ratio^9^ might have a potential predictive role, as in highlighting the situations where immune system is more “receptive” to respond to this therapy.

These observations, if confirmed in larger, possibly prospective series of regorafenib-treated colorectal cancer patients, seem to imply that the identification of some reliable clinical factors such as severe skin rash, diarrhea and hypothyroidism may on the one hand be an early indicator of anti-tumour activity and on the other hand this could lead to an accurate toxicity management aimed at proper symptoms control rather than treatment definitive interruptions^26^. We are currently in the process of designing a trial based upon evaluation of molecular markers that might be relevant to regorafenib methabolism and ultimately might have a role in increasing the toxicity profile of this drug, as to allow a more careful selection of patients where these relevant side effects (diarrhea, hypothyroidism, skin rash) might be considered as surrogates for susceptibility to this drug.

When the balance between toxicity and efficacy is particularly relevant for treatment decision as with regorafenib for metastatic colorectal cancer, patients’ selection becomes crucial^9^. In this setting the possibility to further identify different risk-groups in metastatic colorectal cancer patients represents in fact a key-challenge for the treatment of this disease, particularly now that different options are available and many more are hopefully likely to come.

## Methods

### Patients Selection

All consecutive histologically-proven metastatic colorectal cancer patients, previously treated with standard treatment regimens (oxaliplatin, irinotecan, fluoropyrimidines, bevacizumab and either cetuximab or panitumumab in case of RAS, wild type status) receiving regorafenib monotherapy as per Italian label were eligible for our analysis: in particular, patients included should have received at least 2 prior chemotherapy regimens for metastatic disease, be in a relatively good (ECOG PS: 0–2) performance status, not have any contraindications to regorafenib monotherapy as in the case of active bleeding wounds, uncontrolled high blood pressure or history of recent heart failure or have already received regorafenib in previous lines of treatment. We also included, due to the nature of the analysis, only all those patients who were adequately assessed for regorafenib-based toxicities, while patients where this kind of evaluation was not performed were excluded (it should be noted that no patient under regorafenib treatment was excluded due to the fact that, in Italy, toxicity assessment for regorafenib use is needed to prescribe and administer the drug to a patient).

All patients started treatment with regorafenib at the dose of 160 mg/day (day 1 to 21 each 28-day cycle), dose reductions were applied as clinically indicated. Tumour response was evaluated every 8 weeks by clinicians in accordance to the response evaluation criteria for solid tumours (RECIST version 1.1). Toxicity profile was graded accordingly to the NCI CTCAE criteria (Version 4.0) for all istances where it was possible. In particular, toxicities that were assessed by means of analysis were:

Hand-foot skin reaction (HFSR): patients were considered positive if they developed HFSR greater than or equal to NCI CTCAE grade II, with skin changes and toxicity interfering with ADL.

Skin rash: patients were considered positive if they developed maculopapular rash of intensity greater than or equal to NCI CTCAE grade II, covering 10–30% BSA or more.

Hypertension: patients were considered positive if they developed hypertension greater than or equal to NCI CTCAE grade II, with rounded up values of systolic pressure greater than 150 mmHg and/or values of diastolic pressure greater than 95 mmHg.

Diarrhea: patients were considered positive if they experienced at least one episode of diarrhoea greater than or equal to NCI CTCAE grade II. Also patients who experienced for at least one week during regorafenib intake continued diarrhoea equal in grade to NCI CTCAE grade I were considered positive.

AST/ALT increase: patients were considered positive if they experienced increase in AST/ALT, performed after 4 and after 8 weeks of treatment with regorafenib, greater than or equal to NCI CTCAE grade II, with increase in the range or greater than 3–5 times ULN for the laboratory that performs testing.

Bilirubin increase: patients were considered positive if they experienced increase in bilirubin levels, performed after 4 and after 8 weeks of treatment with regorafenib, greater than or equal to NCI CTCAE grade II, with increase in the range or greater than 1.5–3 times ULN for the laboratory that performs testing.

Hypothyroidism: patients were considered positive if they experienced at least one of the following blood tests alterations performed after 4 and after 8 weeks of treatment with regorafenib, greater than or equal to NCI CTCAE grade I for hypothyroidism. In particular, patients with symptoms of hypothyroidism or even with subclinical hypothyroism that showed increase in TSH levels greater or equal to 2 ULN and/or decrease of fT3/fT4 levels less or equal to 0.5 LLN were considered positive.

### Data Management and statistical Analysis

On the basis of previously published reports we hypothesized that at least 20% of metastatic colorectal cancer patients would experience mild to severe (≥G2 NCI CTCAE) toxicities during regorafenib. Assuming a 75% 6-month OS rate for the group of patients experiencing “severe” (>NCI CTCAE G2) toxicities and a 40% 6-month OS rate for the remaining patients, with α = 0.05 and β = 0.10, 140 patients were needed to confirm our hypothesis (28 patients with ≥G2 NCI CTCAE toxicities and 112 in the remaining group). Statistical analysis was performed with MedCalc Statistical Software version 14.10.2 (MedCalc Software bvba, Ostend, Belgium; http://www.medcalc.org; 2014). The association between categorical variables was estimated by Fisher’s exact test for categorical binomial variables or by chi-square test when indicated. Survival probability over time was estimated by the Kaplan–Meier method. Significant differences in the probability of survival between the strata were evaluated by log-rank test. Cox’s multiple regression analysis was used to assess the role of off-target effects as predictive factors for clinical outcome adjusted for those variables that resulted significant at univariate analysis. The Holm-Sidak correction was used to adjust the values for multiple comparisons. Tested variables included hand-foot skin reaction (HFSR), skin rash, hypertension, diarrhea, fatigue, serum AST/ALT increase, serum bilirubin increase, hypothyroidism. All toxicities were graded as stated before. Other variables analysed were gender (male *vs* female), median age (<70 vs ≥70 years), RAS status (wild type *vs* mutant), ECOG performance status (0 *vs* ≥1), previous lines for metastatic disease (≤3 *vs* ≥4). For statistical analysis, overall survival (OS) was defined as the time interval between the treatment with regorafenib start date and death or last follow-up visit for patients lost at follow-up, whereas progression-free survival (PFS) was defined as the interval between the treatment with regorafenib start date and death, first sign of clinical progression or last follow-up visit for patients lost at follow-up. Disease control rate (DCR) was defined as the sum of the rate of achieving partial or complete response and the rate of patients who obtained stable disease at the first radiological evaluation.

### Ethics

The study was performed in accordance with the protocol, all experimental protocols were approved by the Ethical Committee of the Azienda Ospedaliera Universitaria of Ancona, Italy, written informed consent was obtained for all patients enrolled into the analysis and methods were carried out in accordance with the approved guidelines.

## Additional Information

**How to cite this article:** Giampieri, R. *et al*. Off-target effects and clinical outcome in metastatic colorectal cancer patients receiving regorafenib: The TRIBUTE analysis. *Sci. Rep.* **7**, 45703; doi: 10.1038/srep45703 (2017).

**Publisher's note:** Springer Nature remains neutral with regard to jurisdictional claims in published maps and institutional affiliations.

## Figures and Tables

**Table 1:** Main patients’ characteristics in the overall study population.

Total (%)	
**SEX**	
Male	82 (57)
Female	62 (43)
**Age**	
Median (Range)	62 (32–80)
<70 y.o	123 (86)
≥70 y.o	21 (14)
**RAS status**	
WT	58 (40)
MUT	86 (60)
**Previous anti-EGFR treatment**	58 (40)
**Previous anti-VEGF treatment**	144 (100)
**Previous lines of treatment**	
2	60 (41)
3	62 (43)
4 or more	22 (16)
**ECOG PS at treatment start**	
0	103 (72)
≥1	41 (28)
19	
**Baseline Blood Pressure**	
Normal pressure	62 (43)
Pre-existing hypertension managed by therapy	82 (57)
**Median OS**	6 months
**Median PFS**	2.8 months
**Response Rate**	
PR	9 (7.3)
SD	37 (27)
PD	86 (60)
NE	12 (8)

WT = wild type.

MUT = mutant.

EGFR = Epidermal Growth Factor Receptor.

VEGF = Vascular Endothelial Growth Factor.

ECOG PS = Overall Survival.

OS = Overall Survival.

PFS = Progression-Free Survival.

PR = Partial Remission.

SD = Stable Disease.

PD = Progressive Disease.

NE = Not Evaluated.

**Table 2:** Results of univariate analysis for different regorafenib-related off-target effects on patients’ outcome in terms of DCR, mPFS and mOS.

	DCR	*p*	PFS (months)	HR (95% CI)	*p*	OS (months)	HR (95% CI)	*p*
**HFSR**								
Grade ≥2: 41 (28%)	**47%**	***0.0269***	3.2	0.73 (0.51–1.04)	*0.10*	6.59	0.80 (0.52–1.25)	*0.35*
Grade 0–1: 103 (72%)	**25%**		2.5			5.96		
**SKIN RASH**								
Grade ≥2: 38 (26%)	**55%**	***0.00387***	**3.67**	**0.60 (0.42**–**0.86)**	***0.0096***	**10.36**	**0.54 (0.36**–**0.86)**	***0.0085***
Grade 0–1: 106 (74%)	**21%**		**2.68**			**5.04**		
**HYPERTENSION**								
Grade ≥2: 29 (20%)	48%	*0.0526*	**4.32**	**0.61 (0.41**–**0.89)**	***0.0197***	7.18	0.71 (0.45–1.13)	*0.188*
Grade 0–1: 115 (80%)	27%		**2.65**			5.04		
**FATIGUE Yes/No**								
Grade ≥2: 65 (45%)	32%	*1.0000*	2.85	0.93 (0.66–1.32)	*0.716*	7.18	0.76 (0.50–1.14)	*0.195*
Grade 0–1: 79 (55%)	30%		2.68			6.03		
**AST/ALT ELEVATION**								
Grade ≥2: 18 (12%)	43%	*0.29*	3.01	1.11 (0.65–1.89)	*0.68*	4.16	1.60 (0.83–3.10)	*0.08*
Grade 0–1: 126 (88%)	29%		2.78			6.32		
**BILIRUBIN INCREASE**								
Grade ≥2: 15 (10%)	23%	*0.75*	2.82	1.42 (0.75–2.69)	*0.20*	8.98	1.21 (0.63–2.33)	*0.52*
Grade 0–1: 129 (90%)	32%		2.78			6.03		
**HYPOTHYROIDISM**								
Grade ≥2: 13 (9%)	**71%**	***0.03***	7.96	0.56 (0.34–0.93)	*0.0635*	**NR**	**0.10 (0.05**–**0.19)**	***0.0042***
Grade 0–1: 131 (91%)	**28%**		2.75			**5.83**		
**DIARRHEA**								
Grade ≥2: 21 (15%)	**53%**	***0.0489***	**5.11**	**0.54 (0.36**–**0.81)**	***0.0130***	**11.41**	**0.53 (0.32**–**0.88)**	***0.0440***
Grade 0-1: 123 (85%)	**27%**		**2.65**			**5.27**		

DCR = disease control rate, PFS = progression-free survival, HR = Hazard ratio, CI = confidence interval, OS = overall survival, HFSR = hand and foot skin reaction, AST = Aspartate Aminotransferase, ALT = alanine aminotransferase.
